# Attitudes and opinions towards suicidality in professionals working with oncology patients: results from an online survey

**DOI:** 10.1007/s00520-021-06590-2

**Published:** 2021-10-01

**Authors:** Bianca Senf, Paula Maiwurm, Jens Fettel

**Affiliations:** Department of Psycho-Oncology, University Cancer Center (UCT), Johann Wolfgang Goethe University, Theodor-Stern-Kai 7, 60590 Frankfurt/Main, Germany

**Keywords:** Attitude, Behavior, Cancer, Oncology, Online Survey, Opinion, Suicidality, Understanding

## Abstract

**Objective:**

To explore and describe attitudes and opinions towards suicidality in healthcare professionals (HCPs) working with oncological patients.

**Methods:**

A 48-item online questionnaire was developed and distributed to HCPs working with cancer patients. Three hundred fifty-four answered questionnaires were analyzed.

**Results:**

The majority of HCPs reported that they were able to understand why a cancer patient would commit suicide (87.8%) or would seek help from an assisted suicide organization (ASO; 83.9%). The understandable reasons were pain and physical impairments (51.4%), social isolation (19.8%), loss of control and autonomy (18.1%), terminal disease (17.2%), loss of meaning (15.3%), desperation (14.7%), and psychic distress (9.3%). Personal experiences with suicidality lead only 44.8% of HCPs to believe that thereby they would be better able to understand a patients’ wish for suicide. Religion was negatively associated with understanding of suicide and why a cancer patient would seek help from an ASO. Knowledge of suicidality was positively associated with why a cancer patient would seek help from an ASO.

**Conclusions:**

There is still little knowledge in oncology about the relation of HCPs’ attitudes toward suicidality in their patients and how those attitudes influence their behavior, especially care and treatment of patients. More research on this topic is needed. It stands to reason that more education about suicidality in cancer patients seems likely to improve understanding and attitudes and thereby influence care for cancer patients.

## Introduction

Healthcare professionals (HCPs) working in oncology are time and again exposed to suicidality and even completed suicides in their patients. Although, there is no universally accepted definition for the term suicidality [1–3], in this article, we followed a suggestion defining suicidality in a hierarchy of terms ranging from suicidal ideation, suicidal intention, attempted suicide to completed suicide [2, 3]. Therefore, the term suicidality as used in this article encompasses all forms of suicidal behavior.

It is a well-documented fact that cancer patients have an elevated suicide rate and are at a higher risk for completed suicide than the general population [4–7]. Furthermore, during all stages of the disease trajectory, suicidal crises can develop [8, 9]. An online survey by Senf et al.[9] showed that 83.3% of HCPs working in oncology were confronted with at least one suicidal patient in the year before the survey and 88.1% reported feeling distressed when encountering suicidality in their patients. The main reasons for distress were uncertainty and anxiety (36.6%). A substantial number of HCPs (39.6%) reported a lack of knowledge concerning suicidality and expressed a wish for further education on this topic (81.1%).[9]

Nevertheless, knowledge and education are only two among many factors influencing how HCPs react to suicidal patients. Attitudes influence approach and avoidance behavior and play an important role in processing novel information [10, 11]. For example, attitude conform information is more readily processed that attitude contrary information. This can reduce cognitive dissonance and affect motivation [10]. Motivationally conditioned processing correlates with strength and personal importance of the respective attitude, making attitudes a predictor of behavior. [12] The attitudes HCPs hold towards suicidality and suicidal patients are therefore a crucial factor influencing how HCPs react to suicidality in their patients. Only few studies investigated the attitudes of HCPs towards suicidality in their patients. The majority of studies looked at HCPs working in psychiatry or in emergency departments [13–26]. Only three studies focused on HCPs working in oncology [27–29]. None of those studies investigated the attitude-behavior relationship. Relevant behavior in this case is being treatment and care. Barnfield et al. [13] studied general hospital nurses. Although the nurses reported moderately poor attitudes towards suicide and attempted suicide, e.g., in terms of acceptability and discrimination, they nevertheless believed that they were providing good care to patients that attempted suicide. Among nurses working in emergency departments, higher self-perception of professional competence was associated with less negative feelings towards suicidal patients [19]. Norheim et al. [22] compared HCPs working with suicidal patients in mental health outpatient clinics in Oslo and Stavropol and found overall positive attitudes towards suicidal patients. HCPs in Stavropol agreed that an adequate follow-up should be offered if needed. Psychiatric nurses had a more favorable attitude towards suicide, were more understanding of suicide, and were also more willing to nurse suicidal patients than nurses in general hospitals [25]. These results indicate an association between HCPs attitude and treatment and the care provided to suicidal patients. The nature and extent of this association remains widely unknown as do other factors influencing how HCPs deal with suicidality in cancer patients [27]. Building on the research on exposure to suicidality [9], we focused on the attitudes and opinions of HCPs of different occupational groups working with oncological patients. Attitude towards suicide was conceptualized as the extent to which HCPs are able to understand or relate to why a patient would commit suicide or seek help from an assisted suicide organization (ASO). It is important to have more information on factors influencing how HCPs interact with and react to suicidal patients. This study aimed at closing the existing gaps in knowledge in this area of oncology [27] and thereby contributes to build a groundwork on which to model future educational and preventative strategies that can be employed by HCPs. We particularly explored the following topics:

- How understanding are HCPs of the suicide of a cancer patient?
- Which reasons for suicide seem most understandable to HCPs?
- Have HCPs had any personal experiences with suicidality and does this influence their ability to understand and relate to suicidal patients?
- Is religious affiliation associated with HCPs’ ability to understand and relate to suicidality in their patients?
- Is there an association between HCPs ability to understand and relate to suicidality in their patients and their personal knowledge of and experience with suicidality?
- What are HCPs’ opinions about suicidality?

## Methods

### Study design

We conducted an anonymous online survey over a period of 3 months (December 2017–February 2018). HCPs with patient contact in Germany were invited to answer our questionnaire. The design of this explorative survey was cross-sectional and descriptive.

### Questionnaire

The questionnaire contained 48 items and was developed on the basis of a pre-test among HCPs working with cancer patients. [30] For further information on the questionnaire and the data collection, see Senf et al. [9]

### Data collection

A link to the questionnaire was distributed via the e-mail to HCPs working with cancer patients. At least 150 HCPs should be included. Overall, 1166 survey-link accesses were recorded. Only completely filled questionnaires (*N* = 354) were evaluated. Eight hundred twelve site accesses were not eligible for evaluation because they were not filled in completely or had other internal inconsistencies [9].

### Data analysis

The quantitative data evaluation was performed with the IBM SPSS Version 23.0 Statistics package (IBM Inc., Armonk, New York). The data were descriptively analyzed. Differences between occupational groups were investigated using the Kruskal–Wallis test with subsequent Dunn-Bonferroni test. Metric and ordinal data were analyzed using the Spearman correlation coefficient (*ρ*), and for dichotomous and metric data, the point-biserial correlation coefficient (*r*_*pb*_) was used. Effect strengths were reported according to Cohen’s conventions [31]. Qualitative data were evaluated content-analytically according to Mayring’s method using the software QCAmap [32]. All statements were content analytically categorized and rated by two independent raters. Statements for which no initial agreement could be reached were discussed until a consensus was reached. To assess interrater reliability, Cohen’s-κ was calculated.

### Ethics

The study was carried out in accordance with the ethical standards of the Declaration of Helsinki and received approval from the ethics committee of the University Hospital Frankfurt (ethical approval #20–625). Informed consent was electronically obtained from participants after reading data protection and personal privacy guidelines. All data were collected and stored anonymously.

## Results

### Sample

The data of 354 HCPs working with oncological patients were included. For further information on the sample characteristics, see Table 1 and Senf et al [9].

**Table 1:** Demographic variables

	Total			Physicians		Psychologists		Psychologic Psychotherapists		Nurses			Other		
	*N* = 354 (100%)			*n* = 87 (24.7%)		*n* = 82 (23.2%)		*n* = 59 (16.7%)		*n* = 59 (16.7%)			*n* = 67 (18.9%)		
	*n* (%)	*M* (*SD*) [Min–max]		*n* (%)	*M* (*SD*) [Min–max]	*n* (%)	*M* (*SD*) [Min–max]	*n* (%)	*M* (*SD*) [Min–max]	*n* (%)		*M* (*SD*) [Min–max]	*n* (%)		*M* (*SD*) [Min–max]
**Sex**															
Female	273 (77.1)			45 (51.7)		69 (84.1)		51 (86.4)			51 (86.4)			57 (85.1)	
Male	81 (22.9)			42 (48.3)		13 (15.9)		8 (13.6)			8 (13.6)			10 (14.9)	
**Age (years)**		47.8 (11.5) [23–76]			49.5 (10.6) [27–76]		45.5 (11.5) [26–73]		51.1 (9.9) [29–66]			41 (10.9) [23–61]			51.0 (11.4) [25–75]
**Work with oncological patients (years)**		13.6 (9.3) [1-40]			17.6 (9.8) [1-40]		10.5 (8.5) [1-35]		14.6 (9.3) [1-38]			12.8 (8.4) [1-5]			12.1 (8.9) [1-37]
**Institution**^†^															
Primary care hospital	129 (36.4)			27 (31.0)		35 (42.7)		23 (39.0)		15 (25.4)			29 (44.6)		
Maximum care hospital	121 (34.2)			33 (37.9)		18 (22.0)		9 (15.3)		46 (78.0)			15 (23.1)		
Rehabilitation clinic	29 (8.2)			3 (3.4)		15 (18.3)		7 (11.9)		0 (0.0)			4 (6.2)		
Established panel practice	53 (15.0)			36 (41.4)		2 (2.4)		12 (20.3)		1 (1.7)			2 (3.1)		
Established private practice	38 (10.7)			8 (9.2)		8 (9.8)		13 (22.0)		0 (0.0)			9 (13.8)		
Cancer counseling center	36 (10.2)			1 (1.1)		14 (17.1)		8 (13.6)		0 (0.0)			13 (20.0)		
Outpatient palliative service	7 (2.0)			3 (3.4)		1 (1.2)		0 (0.0)		1 (1.7)			2 (3.1)		
Other	29 (8.2)			0 (0.0)		5 (6.1)		8 (13.6)		2 (3.4)			12 (18.5)		
**Further education**															
Yes, certified by DKG^‡^ (120 h.)	158 (44.6)			23 (26.4)		56 (68.3)		31 (52.5)		3 (5.1)			45 (69.2)		
Yes, certified by DKG^‡^ (100 h.)	31 (8.8)			9 (10.3)		6 (7.3)		14 (23.7)		0 (0.0)			1 (1.5)		
Yes, certified by another Institution	17 (4.8)			4 (4.6)		3 (3.7)		4 (6.8)		4 (6.8)			2 (3.1)		
Yes, non-certified curriculum	31 (8.8)			9 (10.3)		7 (8.5)		5 (8.5)		4 (6.8)					6 (9.2)
Yes, seminar or lecture	19 (5.4)			5 (5.7)		1 (1.2)		0 (0.0)		12 (20.3)			1 (1.5)		
None	98 (27.7)			37 (42.5)		9 (11.0)		5 (8.5)		36 (61.0)			10 (15.4)		
**Religious affiliation**															
No	128 (36.2)		28 (32.2)			31 (37.8)		22 (37.3)		18 (30.5)			27 (41.5)		
Yes	226 (63.8)		59 (67.8)			51 (32.2)		37 (62.7)		41 (69.5)			38 (58.5)		

^†^Multiple answers were possible; the percentage values refer to the number of answers. ‡*DKG*, Deutsche Krebsgesellschaft e.V. (German Cancer Society)

Absolute and relative frequencies of single items concerning attitudes towards suicidality, opinion on suicidality, and personal experience, as well as the results of the omnibus group comparisons, are presented in Table 2. The absolute and relative frequencies of reasons for suicide HCPs which were able to understand and relate to are reported in Table 3. Exemplary statements of understandable reasons for suicide given by HCPs can be found in Table 4.

**Table 2:** Absolute and relative frequencies personal understanding towards suicidality, opinion on suicidality, and personal experience *n* (%) and results of the Kruskal–Wallis-H-test

		Total	Physicians	Psychologists	Psychologic psychotherapists	Nurses	Other	H	***p***
		*N* = 354	*n* = 87	*n* = 82	*n* = 59	*n* = 59	*n* = 65		
**Personal understanding of suicidality**		***N* (%)**				***n* (%)**			
* “How much understanding do you have for the suicide of cancer patients?”*	No understanding at all	0 (0.0)	0 (0.0)	0 (0.0)	0 (0.0)	0 (0.0)	0 (0.0)	7.392	0.117
	Rather no understanding	43 (12.1)	16 (18.4)	7 (8.5)	8 (13.6)	4 (6.8)	8 (12.3)		
	Rather understanding	237 (66.9)	59 (67.8)	58 (70.7)	35 (59.3)	40 (67.8)	45 (69.2)		
	Complete understanding	74 (20.9)	12 (13.8)	17 (20.7)	16 (27.1)	15 (25.4)	12 (18.5)		
* “How much understanding do you have for the suicide of non-somatically ill patients?”*	No understanding at all	10 (2.8)	2 (2.3)	1 (1.2)	0 (0.0)	4 (6.8)	3 (4.6)	5.370	0.251
	Rather no understanding	136 (38.4)	30 (34.5)	29 (35.4)	24 (40.7)	29 (49.2)	24 (36.9)		
	Rather understanding	177 (50.0)	47 (54.0)	45 (54.9)	32 (54.2)	20 (33.9)	31 (47.7)		
	Complete understanding	31 (8.8)	8 (9.2)	7 (8.5)	3 (5.1)	6 (10.2)	7 (10.8)		
* “How great is your understanding of the wish of patients to make use of the services of a euthanasia organization?”*	No understanding at all	4 (1.1)	2 (2.3)	1 (1.2)	0 (0.0)	1 (1.7)	0 (0.0)	12.660	0.013
	Rather no understanding	53 (15.0)	20 (23.0)	8 (9.8)	7 (11.9)	6 (10.2)	12 (18.5)		
	Rather understanding	193 (54.5)	49 (56.3)	48 (58.5)	30 (50.8)	30 (50.8)	34 (52.3)		
	Complete understanding	104 (29.4)	16 (18.4)	25 (30.5)	22 (37.3)	22 (37.3)	19 (29.2)		
**Opinion on suicidality**									
* “Most patients who commit suicide suffer from depression.”*	I don’t agree at all	14 (4.0)	3 (3.4)	3 (3.7)	4 (6.8)	1 (1.7)	3 (4.6)	2.123	0.713
	I rather not agree	115 (32.5)	26 (29.9)	31 (37.8)	14 (23.7)	22 (37.3)	22 (33.8)		
	I rather agree	212 (59.9)	58 (66.7)	48 (58.5)	39 (66.1)	32 (54.2)	33 (50.8)		
	I agree completely	13 (3.7)	0 (0.0)	0 (0.0)	2 (3.4)	4 (6.8)	7 (10.8)		
* “Most patients who commit suicide suffer from some kind of mental disorder.”*	I don’t agree at all	38 (10.7)	8 (9.2)	8 (9.8)	4 (6.8)	10 (16.9)	8 (12.3)	14.849	0.005
	I rather not agree	171 (48.3)	45 (51.7)	36 (43.9)	21 (35.6)	36 (61.0)	33 (50.8)		
	I rather agree	136 (38.4)	33 (37.9)	38 (46.3)	32 (54.2)	10 (16.9)	21 (32.3)		
	I agree completely	9 (2.5)	1 (1.1)	0 (0.0)	2 (3.4)	3 (5.1)	3 (4.6)		
* “If an oncological patient aborts or ceases his/her therapy, that is a sign for suicidality.”*	I don’t agree at all	145 (41.0)	33 (37.9)	35 (42.7)	26 (44.1)	21 (35.6)	30 (46.2)	2.686	0.612
	I rather not agree	189 (53.4)	46 (52.9)	44 (53.7)	29 (49.2)	35 (59.3)	33 (50.8)		
	I rather agree	18 (5.1)	7 (8.0)	3 (3.7)	4 (6.8)	3 (5.1)	1 (1.5)		
	I agree completely	2 (0.6)	1 (1.1)	0 (0.0)	0 (0.0)	0 (0.0)	1 (1.5)		
* “Most suicidal oncological patients lack fighting spirit.”*	I don’t agree at all	174 (49.2)	44 (50.6)	44 (53.7)	35 (59.3)	23 (39.0)	28 (43.1)	8.293	0.081
	I rather not agree	155 (43.8)	36 (41.4)	36 (43.9)	22 (37.3)	30 (50.8)	30 (46.2)		
	I rather agree	22 (6.2)	6 (6.9)	2 (2.4)	2 (3.4)	6 (10.2)	5 (7.7)		
	I agree completely	3 (0.8)	1 (1.1)	0 (0.0)	0 (0.0)	0 (0.0)	2 (3.1)		
* “For most oncological patients suicidality is not an issue.”*	I don’t agree at all	67 (18.9)	14 (16.1)	22 (26.8)	14 (23.7)	10 (16.9)	7 (10.8)	12.854	0.012
	I rather not agree	141 (39.8)	34 (39.1)	24 (29.3)	23 (39.0)	36 (61.0)	22 (33.8)		
	I rather agree	129 (36.4)	36 (41.4)	33 (40.2)	19 (32.2)	12 (20.3)	29 (44.6)		
	I agree completely	17 (4.8)	3 (3.4)	3 (3.7)	3 (5.1)	1 (1.7)	7 (10.8)		
* “For oncological patients suicidal thoughts are a means for regaining control.”*	I don’t agree at all	22 (6.2)	6 (6.9)	7 (8.5)	1 (1.7)	3 (5.1)	5 (7.7)	26.226	< 0.001
	I rather not agree	74 (20.9)	14 (16.1)	11 (13.4)	6 (10.2)	28 (47.5)	15 (23.1)		
	I rather agree	223 (63.0)	61 (70.1)	51 (62.2)	43 (72.9)	26 (44.1)	40 (61.5)		
	I agree completely	35 (9.9)	6 (6.9)	13 (15.9)	9 (15.3)	2 (3.4)	5 (7.7)		
* “Most patients that announce a suicide do not follow through.”*	I don’t agree at all	68 (19.2)	11 (12.6)	24 (29.3)	21 (35.6)	3 (5.1)	9 (13.8)	18.396	0.001
	I rather not agree	172 (48.6)	43 (49.4)	34 (41.5)	26 (44.1)	34 (57.6)	33 (50.8)		
	I rather agree	107 (30.2)	32 (36.8)	23 (28.0)	11 (18.6)	21 (35.6)	20 (30.8)		
	I agree completely	7 (2.0)	1 (1.1)	1 (1.2)	1 (1.7)	1 (1.7)	3 (4.6)		
**Personal experience**									
* “Have you already made experiences with suicidality in your personal environment?”*	Yes	201 (56.8)	47 (54.0)	46 (56.1)	41 (69.5)	25 (42.4)	40 (61.5)	NA	NA
	No	153 (43.2)	40 (46.0)	36 (43.9)	18 (30.5)	34 (57.6)	25 (38.5)		
* “Do you believe that you can better understand the suicide wish of patients based on your own personal experience with the subject?”*	Yes	90 (25.4)	21 (24.1)	23 (28.0)	18 (30.5)	9 (15.3)	17 (26.2)	NA	NA
	No	111 (31.4)	26 (29.9)	23 (28.0)	23 (39.0)	16 (27.1)	23 (35.4)

**Table 3:** Reasons for suicide HCPs were able to understand/relate to (categorized; results of qualitative evaluation, multiple answers were possible)

	Total		Physicians		Psychologists		Psychologic Psychotherapists		Nurses		Other	
	*N* = 354		*n* = 87		*n* = 82		*n* = 59		*n* = 59		*n* = 65	
Pain and other severe physical impairments	182	(51.4%)	43	(49.4%)	42	(51.2%)	39	(66.1%)	26	(44.1%)	32	(49.2%)
Social isolation	70	(19.8%)	13	(14.9%)	15	(18.3%)	15	(25.4%)	14	(23.7%)	13	(20.0%)
Loss of control and autonomy	64	(18.1%)	18	(20.7%)	18	(21.9%)	15	(25.4%)	5	(8.5%)	8	(12.3%)
Terminal disease	61	(17.2%)	9	(10.3%)	13	(15.9%)	8	(13.6%)	18	(30.5%)	13	(20.0%)
Desperation	52	(14.7%)	15	(17.2%)	10	(12.2%)	10	(16.9%)	7	(11.9%)	10	(15.4%)
Loss of meaning	54	(15.3%)	9	(10.3%)	20	(24.4%)	10	(16.9%)	7	(11.9%)	8	(12.2%)
Anxiety, depression, and other psychic distress	33	(9.3%)	7	(8.0%)	12	(14.6%)	3	(5.1%)	3	(5.1%)	8	(12.3%)
Avoiding pain for others	4	(1.1%)	1	(1.1%)	0	(0.0%)	0	(0.0%)	3	(5.1%)	0	(0.0%)
Unrelatable	4	(1.1%)	0	(0.0%)	1	(1.2%)	1	(1.7%)	1	(1.7%)	1	(1.5%)

**Table 4:** Exemplary statements of understandable reasons for suicide given by HCPs

**Category**	**Example**”I am able to relate to … as reason for suicide.”
Pain and other severe physical impairments	*…non-treatable pain…* *…constant dyspnea… / …fear of suffocating…* *…prospect of a life with disabilities…*
Social isolation	*…no family… / …no friends… / …non supportive family…* *…single patient/ loneliness…* *…financial difficulties and thereby not being able to pay for further therapy…*
Loss of control and autonomy	*…loss of control over one’s own life… / …being totally dependent on others…* *…wish for control in a seemingly desperate situation… / …wish for a self-determined death…*
Terminal disease	*…imminent and certain end of life…* *…terminal palliative condition…*
Desperation	*…absolute desperation…* *…being helplessness and impotent…*
Loss of meaning	*…extended suffering…* *…severely impaired quality of life…* *…loss of dignity…*
Anxiety, depression, and other psychic distress	*…severe depression, psychosis, severe psychic distress…* *…loss of sleep and anxiety…*
Avoiding pain for others	*…being a burden and pain for relatives…*
Non-understandable	*There are no reasons*

### Being able to understand a patient’s suicide (Table 2)

The majority of HCPs (87.8%) reported being able to understand why a cancer patient would commit suicide. There was no significant difference between occupational groups.

Over half of HCPs (58.8%) reported being able to understand why non-somatically ill patient would commit suicide. There was no difference between occupational groups.

A total of 83.9% of HCPs stated being able to relate to a patients’ wish for seeking help from an ASO. A significant difference between occupational groups was found (*H*_*(4)*_ = 12.660, *p* = 0.013): Physicians (74.4%) were significantly less understanding than nurses (88.1%) (*Z* =  − 2.87, *p* = 0.041, *r* =  − 0.24) and psychologic psychotherapists (88.1%) (*Z* =  − 2.91, *p* = 0.037, *r* =  − 0.24).

### Reasons for suicide (Table 3)

Of the 354 participants, 333 gave valid statements. Overall, *N* = 750 valid statements were rated. The agreement rate was 76.67%, with Cohen’s-κ = 0.73, *p* < 0.001 which translates to a substantial interrater agreement [33].

The ranking of understandable reasons for suicide (Table 3) was pain and other severe physical impairments (51.4%), social isolation (19.8%), loss of control and autonomy (18.1%), terminal disease (17.2%), loss of meaning (15.3%), desperation (14.7%), anxiety, depression and other psychic distress (9.3%), and finally avoiding pain for others (1.1%). Only 1.1% reported no understandable reasons for suicide.

### Personal experiences with suicidality (Table 2)

In total, 56.8% of HCPs had already experiences with suicides in their personal environment.

With the exception of nurses (42.4%), all occupational groups over half of participants had already experiences with suicides in their personal environment (physicians = 54.0%, psychologists = 56.1%, psychologic psychotherapists = 69.5%, others = 61.5%).

Of those who reported having had experience with suicides (*n* = 201), 44.8% believed that thereby they would be better able to understand a patients’ wish for suicide.

This pattern seems to be the same in most occupational groups (physicians = 44.7%, psychologic psychotherapists = 43.9%, others = 42.5%) with the exception of nurses (36.0%) and psychologists (50.0%).

### Relation between religious affiliation and understanding of suicide

About two thirds of HCPs (63.8%) were somehow religiously affiliated. There was no difference between occupational groups (*χ*^*2*^_*(4)*_ = 2.345, *p* = 0.673). Religious affiliation was negatively associated with being able to understand why a cancer patient would commit suicide (*r*_*pb*_ =  − 0.163, *p* = 0.002) and understanding a patients’ wish of seeking help from an ASO (*r*_*pb*_ =  − 0.149, *p* = 0.005).

### Relation between being able to understand a patient’s suicidality and personal knowledge/experience about suicidality

HCPs self-rated knowledge of facts about suicidality in cancer patients correlated with being able to understand a patients’ wish of seeking help from an ASO (*ρ* = 0.11, *p* = 0.039).

### HCPs opinion about suicidality (Table 2)

In total, 63.6% of HCPs agreed with the statement that most patients that commit suicide are depressed. There were no significant differences between occupational groups.

Overall, 59.0% of HCPs disagreed with the statement that most patients committing suicide have some kind of mental disorder. The occupational group that mostly disagreed were nurses (77.9%). Of the psychologic psychotherapists instead, only 42.4% disagreed. There was a significant difference between occupational groups (*H*_*(4)*_ = 14.849, *p* = 0.005): psychologic psychotherapists disagreed less with this statement than nurses (*Z* = 3.73, *p* = 0.002, *r* = 0.34).

Nearly all HCPs (94.4%) disagreed with the statement that a cancer patient discontinuing therapy is an indication for suicidality. No significant difference between occupational groups was found.

The majority (92.9%) disagreed with the statement that suicidal cancer patients have no fighting spirit. No significant difference between occupational groups was found.

That suicide is not an issue for most oncological patients was also disagreed with by the majority of HCPs (58.8%). Especially nurses (78.0%) and psychologic psychotherapists (62.7%) disagreed, less so psychologists (56.1%), physicians (55.2%), and others (44.6%). There was a difference between occupational groups (*H*_*(4)*_ = 12.854, *p* = 0.012) in that nurses disagreed significantly more than others (*Z* =  − 3.36, *p* = 0.008, *r* =  − 0.30). No other difference between any occupational groups was significant.

The overall agreement with the statement that suicidal thoughts are a means of regaining control for cancer patients was 72.9%. There was a difference between occupational groups (*H*_*(4)*_ = 26.226, *p* < 0.001): Nurses (69.2%) agreed less with this statement than physicians (77.0%) (*Z* = 3.19, *p* = 0.014, *r* = 0.26), psychologists (78.1%) (*Z* = 3.97, *p* = 0.001, *r* = 0.33), and psychologic psychotherapists (88.2%) (*Z* = 4.76, *p* < 0.001, *r* = 0.44).

Overall, 67.8% of HCPs disagreed with the statement that most patients who announce a suicide do not follow through. There was a difference between occupational groups (*H*_*(4)*_ = 18.396, *p* = 0.001): Psychologic psychotherapists (70.8%) disagreed significantly more than others (64.6%) (*Z* =  − 2.87, *p* = 0.042, *r* =  − 0.26), physicians (37.9%) (*Z* = 3.24, *p* = 0.012, *r* = 0.27), and nurses (37.3%) (*Z* =  − 3.46, *p* = 0.005, *r* =  − 0.32).

## Discussion

We investigated HCPs attitudes and opinions towards suicidality. We looked at factors that influence attitudes and whether attitudes affect how HCPs approach patients. We hope this might contribute to closing the gaps concerning the knowledge of suicidality in oncology [27]. The results are meant to complement the findings regarding exposure to suicidality in HCPs working with cancer patients [9].

The majority of HCPs are able to understand the wish of cancer patients to die or seek help from an ASO under certain circumstances. HCPs have less understanding of suicidality in non-somatically ill patients. This corresponds with previous research that suicides by patients with physical illness were more acceptable than suicides by psychiatric patients [28]. In our study, nurses and psychologists were most understanding whereas physicians were least understanding. Contrary, the HCPs investigated by Grimholt et al.[20] had only a slight understanding of the wish of patients with incurable disease to end their life. They disagreed with the statement that patients should be supported, if they asked for help ending their life. This is not directly comparable with the item we employed, because we asked for HCPs’ understanding for involving an ASO and not the HCPs themselves. Physician-assisted suicide (PAS) has gained public acceptance over the last decades [34]. But there seems to be less acceptance of PAS by physicians themselves, particularly palliative care specialists [35]. Rejection of PAS correlated with a higher level of qualification in the field of palliative care [35]. Nevertheless, when physicians were asked about their attitudes if they themselves were the patient, support for PAS increased. This hints at the role of attitudes in influencing behavior. There has been little research into the reasons why physicians reject PAS. Religion was identified as a factor associated with opposition to PAS [34]. A debate on the topic from a secular point of view produced four arguments against PAS: it devalues life, it constitutes “slippery slope” (limits of PAS are gradually being eroded), modern palliative medicine can manage pain, and it is undermining the physicians’ integrity and violates patient trust [36]. Concerning religious affiliation of HCPs, we found slightly negative correlations between religious affiliation and understanding of suicide and seeking help from an ASO. Nurses without religion had more positive attitudes towards suicidal behavior than those with a religion [26]. A study differentiating further by confession found that protestants and regular church goers had more condemnatory attitudes [14]. The considerable impact of religion on attitudes towards suicide on a societal and individual level has been shown in international studies [37, 38].

The reasons for suicidality HCPs can most relate to are foremost of somatic origin. Somatic reasons (pain, physical condition) constitute over one third of statements followed by a wide margin by social and existential reasons (social isolation and loss of control and autonomy) and only then psychic reasons (desperation). This pattern varies only slightly over occupational groups. Only psychologic psychotherapists (16.9%) cited desperation among the top three reasons. A study exploring HCPs’ perspectives of their patient’s mental health distress discovered a similar pattern of reasons: diseases related, social, and existential factors [39]. A further study investigated how HCPs perceive suicidality in patients with cancer [27]. HCPs explanatory models of suicide in their patients can be grouped into four categories: biological disease, mental illness, aberration, and impulsive act. Although it was not specifically investigated in this study, it seems likely that understandable reasons for suicide given by HCPs are associated with the attitudes they hold [27].

It seems reasonable to assume that personal experiences with suicide would have an impact on HCPs own attitudes. A study with psychiatric patients yielded no clear results [40]. Unfortunately, research in this area, especially in oncology, is lacking. We found that having had experiences with suicidality in the personal environment made it easier for some HCPs to understand suicidality in their patients. This replicates the finding that doctors who themselves ever contemplated suicide or had a relationship with a person who committed suicide had more positive attitudes towards patients who attempted suicide [23]. Mostly psychologists believed that they would be better able to understand a patients’ wish for suicide when they had already personal experiences with suicide. Nurses were least convinced that would be an influencing factor. Whether those differences between occupational groups are systematic is unclear. Here also more research is needed.

Opinions are commonly conceptualized as the cognitive component of attitudes and constitute the building blocks of attitudes [10]. It is important to explore which opinions concerning suicidality in cancer patients are relevant to HCPs. This was explored by Granek et al., but they did not differentiate between attitudes and opinions/believes [27]. Our results show that the majority of HCPs hold beliefs that are grounded in clinical and empirical reality and reflect the current evidence-based state of science. About two-thirds of HCPs believed that most patients that commit suicide are depressed. Depression and other mood disorders are strong predictors for suicide and suicidal behavior [41]. Epidemiological studies show that about 40 to 60% of people who committed suicide were suffering from depression at the time of their suicide [42]. It might be that most HCPs in our sample are aware of those facts and therefore mostly agreed with this statement. Whether this belief might also lead to more awareness of depressive symptoms as a possible risk factor of suicidality is unclear. In contrast with this finding, over half of HCPs rejected the statement that most patients committing suicide have some kind of mental disorder. This is puzzling, because depression is also a mental disorder. Only psychologic psychotherapists thought otherwise, maybe because of their specific expertise in this field. This discrepancy might also have to do with the wording of this second statement, in that depression in everyday parlance might not necessarily be that closely associated with mental illness, and on the other hand, mental disorder has often a very stigmatizing ring to it. Most interestingly nearly three-fourths of HCPs believed that suicidal thoughts can be a means for cancer patients to regain control. Only nurses did mostly disagree. This aspect is of special importance, because here cancer patients or patients with somatic diseases in general differ fundamentally from psychiatric patients. Starting with the diagnosis and onward through treatment and therapy, patients report situations in which they feel helpless and without control [43, 44]. In a sense, the ultimate means to regain control for the patient in those difficult situations can be being aware that one still retains the power to end it all if things take an even further turn for the worse [45]. The buffering effect of sense of control against symptoms of distress suggests that a higher sense of control helps in remaining engaged in social life and thereby mitigating anxiety [46]. Furthermore, patients with a strong sense of control over life reported less anxiety and worries [47]. Being aware of the fact that suicidal ideations can serve the effort to regain control might be an important skill of HCPs working with cancer patients. It might help them to better understand the current state a patient is in and enable them to direct prevention efforts at patients who actually need them. Finally, over two-thirds of HCPs disagreed with the statement that patients who announce suicide do not actually commit suicide. Especially psychologic psychotherapists and psychologists did not agree which might be again due to their greater experience in this area. In the year prior to suicide, on average, 80% of patients who committed suicide had contact with primary healthcare and even just 1 month prior the contact rate was still 44% [48]. Additionally, patients who committed suicide had more contacts with hospital doctors and social workers [49]. Even if general practitioners identified suicidality in their patients, they often seemed unable to go beyond assessment and did not directly address the topic [50]. To our knowledge, no research exists concerning this topic in oncology. Therefore, it is important for oncology HCPs to take suicidality in their patients seriously and have the special knowledge and assessment skills to refer patients to specialized HCPs.

### Clinical implications

The lack of research on the topic of HCPs’ attitudes and opinions on suicidality in their patients makes suggesting concrete clinical interventions difficult and maybe premature. We think that the first course of action should be to gather more information on those topics and build a solid base of knowledge that can support evidence-based suggestions. This means that more research in this area at the intersection of suicidology and oncology is necessary. For example, a consensus definition on what is meant by suicidality, suicide, and associated phenomena like PAS or euthanasia would be needed to ensure comparability of research. Similarly, the assessment of attitudes and opinions needs to be based on an agreed upon framework. Proceeding from there, the formation and maintenance of attitudes could be investigated, taking cultural and legal differences in different countries into consideration.

Other areas of psycho-oncology (e.g., implementation of distress screenings) can give an indication of possible measures that could be employed in the meantime until more knowledge becomes available. We know, for example, that a communication training for HCPs has beneficial effects on communication behavior as well as attitudes [51]. Addressing the topic of suicidality in patient consultations can help patients and HCPs find a constructive way of dealing with this issue should it arise. Riedl and Schüßler[52] could show that an improved patient-doctor communication has positive effects in patients’ health and compliance. Formal and informal education about suicidality can mitigate or even overcome misunderstandings and myths concerning this topic [53]. As these misconceptions and myths often have their basis in the distortion of facts and half-truths, thusly contributing to the stigmatization of suicidal individuals [54], conveying truthful information to HCPs should be the best way of addressing this issue [53, 54]. The evaluation of the KoMPASS-program, a comprehensive communication training for oncologists, shows how such a training could be set up. Role-playing, video feedback, and joint discussions of individual cases were rated especially helpful. The theoretical part of the program was also positively evaluated, showing the importance of joining practical and theoretical elements [55].

### Limitations

There are certain limitations to this study. The study sample limits the generalizability of the results. Participation in an online survey introduces selection bias exists due to the voluntary nature of participation. Therefore, generalizability beyond the study population cannot be assumed. Due to using an online questionnaire and the method of recruitment, it was not possible to calculate an exact response rate. The nature of the topic may have led some participants to answer more socially desirable or otherwise according to cultural or societal norms of their specific subpopulation. The assessment of attitudes towards suicidality could be improved and more specific than the generic understanding of certain patient behaviors. The topic of religion could be expounded in more detail by asking for confessions a more detailed picture could be developed.

## Conclusions

There is little research on the attitudes of HCPs towards suicidality in their patients and even less on how attitudes influence patient treatment and care. Therefore, we think that more basic research on HCPs’ attitudes toward suicidality in cancer patients is necessary. If, as we assume, decades of research show [10, 12] strong attitudes influence and are predictive of behavior, it is important to know which attitudes exist among HCPs, by what factors they are determined, and how they exert their influence on behavior, especially treatment and care. A further step would be whether dysfunctional attitudes can be influenced or even changed, not only to improve care and treatment of cancer patients, but to achieve better working conditions for the HCPs themselves.

Suicidality in patients can be very upsetting and disturbing for HCPs and lead to ignorance and avoidance. As the results of Senf et al. indicate, ignorance and avoidance are often fueled by feeling insecure and uncertain about how to best deal with suicidality and the fear of making mistakes [9]. Multiple studies indicate an association between the valence of attitudes toward suicidality and experience and knowledge HCPs have with this topic. For example, mental health nurses, who have more experience and knowledge with suicidal patients, tend to hold more positive attitudes towards them [17, 18]. Furthermore, more positive attitudes are also associated with feeling more confident and competent treating suicidal patients [19, 21]. Negative attitudes can lead to a lack of empathy and stigmatization, resulting in a decreased quality of care [24]. More experience with suicidal patients and more knowledge were related to more positive attitudes, indicating that education on the topic cloud be an important factor in positively influencing attitudes [26]. In a study by Briggs, 40% of participants specifically requested education and training [16]. This is in line with Senf et al. where more than 80% of HCPs wanted further education on suicidality in cancer patients [9]. More education about suicidality in cancer patients seems likely to improve understanding and attitudes and thereby have an impetus on how HCPs care for cancer patients.

## Data Availability

The data that support the findings of this study are available on request from the corresponding author. The data are not publicly available due to privacy or ethical restrictions.

## Code availability

N/A.
